# Arabidopsis IAR4 Modulates Primary Root Growth Under Salt Stress Through ROS-Mediated Modulation of Auxin Distribution

**DOI:** 10.3389/fpls.2019.00522

**Published:** 2019-04-25

**Authors:** Yang Fu, Yong Yang, Shaoping Chen, Nina Ning, Honghong Hu

**Affiliations:** National Key Laboratory of Crop Genetic Improvement, College of Life Science and Technology, Huazhong Agricultural University, Wuhan, China

**Keywords:** *IAR4*, salt stress, primary root growth, ROS, auxin transport, root meristem activity

## Abstract

High salinity is one of the major environmental stresses that plants encounter. Roots are the initial and direct organs to perceive the signal. However, how plant roots perceive and respond to salinity at the molecular and physiological levels is still poorly understood. Here, we report that *IAA-CONJUGATE-RESISTANT 4* (*IAR4*) plays a key role in primary root growth under salt stress conditions. Mutation of *IAR4* led to increased sensitivity to salt stress conditions, with strongly inhibited primary root growth and reduced survival rate in two *iar4* mutant alleles. *iar4* mutants accumulated greater Na^+^ and exhibited a greater Na^+^/K^+^ ratio under NaCl treatment. In addition, more reactive oxygen species (ROS) accumulated in the *iar4* mutants due to reduced ROS scavenging. NaCl treatment greatly suppressed the expression levels of *ProPIN1:PIN1-GFP*, *ProPIN2:PIN2-GFP*, *ProPIN3:PIN3-GFP*, and *ProDR5:GFP*, and suppressed root meristem activity in *iar4*. GSH or auxin treatment greatly recovered the *PIN* expression, auxin distribution and primary root growth in the *iar4* mutants, suggesting ROS is a vital mediator between salt stress and auxin response. Our data support a model in which IAR4 integrates ROS and auxin pathways to modulate primary root growth under salinity stress conditions, by regulation of PIN-mediated auxin transport.

## Introduction

Salinity is one of the key environmental factors that seriously affects plant growth and development, and thus influences crop production worldwide (Zhu, 2002; Munns and Tester, 2008). Among all types of salts in soil, sodium chloride is the most soluble and widespread, exerting both osmotic stress and ionic stress on plants (Hasegawa et al., 2000; Hernandez et al., 2001; Zhu et al., 2007; Munns and Tester, 2008; Yu et al., 2010). The root is the first and direct organ to sense elevated salinity levels (Wang et al., 2009). Previous reports show that salt stress represses root meristem activity, root cell cycle, and cell elongation (Burssens et al., 2000; West et al., 2004; Bustos et al., 2008; Jiang et al., 2016), resulting in primary root growth retardation (Bustos et al., 2008; Liu et al., 2015; Jiang et al., 2016). In addition, salt stress also affects root gravitropism (Sun et al., 2008) and lateral root formation (Wang et al., 2009; Zolla et al., 2010).

Auxin plays an essential role in root meristem maintenance and root growth (Han et al., 2017), depending on auxin levels and distribution in root tips, where auxin maximum is formed and maintained by polar auxin transport (Sabatini et al., 1999; Blilou et al., 2005; Dello Ioio et al., 2008; Yuan et al., 2013, 2014; Hong et al., 2014; Mroue et al., 2018). It was reported that salt stress markedly disturbed auxin homeostasis and distribution patterns in primary roots (Wang et al., 2009), which suppressed the expression of the auxin carrier *PIN-FORMED 2* (*PIN2*), and repressed the stabilization abundance of the PIN2 protein and INDOLE-3-ACETIC ACID INDUCIBLE 17 (IAA17), thereby repressing auxin signaling and root meristem development (Sun et al., 2008). A recent study showed that salt stress induced *MIR393A* expression and led to a reduction of TRANSPORT INHIBITOR RESPONSE 1 (TIR1) and AUXIN SIGNALING F-BOX 2 (AFB2) receptors, which subsequently triggered stabilization of the Aux/IAA repressors and led to the decline of auxin signaling (Iglesias et al., 2014). These results suggest that auxin distribution and signaling mediate plant response to salt stress conditions.

An elevated Na^+^ level breaks the balance between ROS (reactive oxygen species) production and scavenging, causing ROS accumulation (Miller et al., 2010; Ma et al., 2012), which subsequently results in oxidative stress and cell damage (Ling et al., 2009; Miller et al., 2010). Meanwhile, ROS also serves as a vital second messenger that regulates the balance between cellular proliferation and differentiation in roots, and modulates root growth and development (Shoresh et al., 2011; He et al., 2012).

Reactive oxygen species and auxin are two key switch elements that are used by plants to trigger dynamic responses to salt stress conditions (Chen and Miller, 2014). The crosstalk between auxin and ROS has gained attention in terms of plant growth and development. ABA-induced ROS production in mitochondria can control *PLETHORA* (*PLT*) expression and auxin accumulation to regulate root meristem activity (Yang et al., 2014). On the other hand, auxin can regulate ASCORBATE PEROXIDASE 1 (APX1) activity to induce redox change, causing alteration of root growth and development (Correaaragunde et al., 2013; Chen and Miller, 2014). An auxin receptor mutant *tir1afb2* showed enhanced tolerance to salt stress with enhanced redox metabolism, also supporting the opinion that auxin regulates ROS status (Iglesias et al., 2010). However, how salt stress and root development are integrated by ROS-auxin crosstalk is still not clear.

To identify genes that function in salt stress and to understand the relationship between salt stress and auxin response, we focused on salt-inducible and auxin-responsive genes according to their expression patterns using Gene Investigator. We collected around 100 T-DNA insertion lines of these genes. Through screening the growth performance under salt stress conditions, we identified that *IAR4* was involved in salt-inhibited primary root growth. *iar4* mutants showed hypersensitive response to salt stress and primary root growth retardation. In addition, salt stress induced ROS overaccumulation in the *iar4* mutants by impairing ROS scavenging. Salt stress greatly suppressed the expression levels of *ProPIN1:PIN1-GFP*, *ProPIN2:PIN2-GFP*, *ProPIN3:PIN3-GFP*, and *ProDR5:GFP* and suppressed root meristem activity in the *iar4* mutants, which could be largely rescued by glutathione (GSH) antioxidant or auxin treatment. Taken together, our study provides a mechanistic understanding of how *IAR4* mediates the root response to salt stress and illustrates the potential role between ROS and auxin in primary root growth.

## Materials and Methods

### Plant Materials and Growth Conditions

All Arabidopsis lines used in this study were in the Columbia background (Col-0). The *IAR4* mutant line *iar4-7* (SALK_091909) was obtained from the Arabidopsis Biological Resource Center (ABRC) and *iar4-8* (SALK_137286) was obtained from Dr. Lin Xu (Institute of Plant Physiology and Ecology, SIBS, CAS). Plants were grown on Murashige and Skoog (MS) medium containing different supplements in a Percival chamber with controlled conditions (22°C, 16 h light/8 h night regime, 80 μmol photons m^-2^ s^-1^ light intensity, and 70% relative humidity).

### Salt Tolerance and Phenotype Identification

To determine the survival rate under salt stress conditions, at least 50 seeds of the wild-type (WT) and *iar4-7* mutant per experiment were sown on MS medium supplemented with 150 mM NaCl for 3 weeks. For determination of primary root growth under salt or osmotic stress conditions, 3-day-old *iar4-7*, *iar4-8*, and WT seedlings were grown on vertical MS medium plates, and were transferred to MS medium supplemented with NaCl (0, 50, 75, 100, 120 mM) or mannitol (0, 100, 150, 200 mM) and incubated vertically for 5 days. For the effect of GSH or auxin on primary root growth suppressed by NaCl, 3-day-old seedlings on MS medium were transferred to MS medium supplemented with 100 mM NaCl, 100 mM NaCl plus 200 mM GSH, 100 mM NaCl plus 0.1 μM IAA, or 100 mM NaCl plus 0.1 μM NAA for 10 days. All experiments were repeated three times, and the error bars indicated the standard deviation (SD).

### Creation of the Complementation Construct and Transgenic Plants

For the complementation assay, the genomic DNA fragment containing the complete *IAR4* gene and native promoter was amplified by the primer pairs listed in Supplementary Table S1. The purified PCR product was digested with *Apa*I and *Spe*I, and then was cloned into the corresponding sites of the pGPTVII-hyg binary vector (Walter et al., 2004). The *ProIAR4:IAR4* construct was transformed into *iar4-7* via the floral dipping method (Clough and Bent, 1998). The transgenic plants were screened on MS medium containing 25 mg L^-1^ hygromycin. Three randomly selected independent transgenic T3 lines (COM-1, COM-3, COM-5) were used for further experiments.

### RT-PCR and qRT-PCR Analyses

Semi-quantitative real-time PCR (RT-PCR) was used to detect *IAR4* expression in the leaves of WT, *iar4-7*, *iar4-8*, and *IAR4* complementation plants. qRT-PCR was used to detect the expression levels of salt-responsive genes and ROS-related genes in 7-day-old WT and *iar4-7* seedlings that treated with 0 or 100 mM NaCl for 8 h. Total RNAs were extracted using TRIzol reagent (Ambion), and the first-strand cDNAs were synthesized using M-MLV Reverse Transcriptase (Madison, WI, United States) according to the provided protocols. *Actin7* was used as an internal control for RT-PCR and *UBQ5* was used as an internal control for qRT-PCR. qRT-PCR was performed with the Bio-Rad CFX96 Real-Time System using Universal SYBR Green Supermix (Bio-Rad). The PCR amplification was performed with the procedure of 1 min at 95°C, 50 cycles of 10 s at 95°C, 15 s at 55–60°C and 15 s at 72°C, 5 min at 72°C. The primer sequences used for RT-PCR and qRT-PCR were listed in Supplementary Table S1.

### Determination of ROS Levels

H_2_O_2_ levels in roots were detected by 2′,7′-dichlorodihydrofluoresin diacetate (H_2_DCF-DA) (Sigma-Aldrich) and 3′,3′-diaminobenzidine (DAB) staining. For H_2_DCF-DA staining, 5-day-old *iar4-7*, *iar4-8*, and WT seedlings grown on MS medium were treated with 100 mM NaCl for 30 min, and then their roots were immersed in 20 μm H_2_DCF-DA buffer for 20 min. A laser scanning confocal (TCS SP8, Leica) microscope was used to detect the fluorescence with excitation at 488 nm and emission at 525 nm. The relative fluorescence was analyzed with ImageJ software, and the fluorescence in control plants without treatment was set as 100%. H_2_O_2_ detection by 3′,3′-diaminobenzidine (DAB, 1 mg mL^-1^) staining and superoxide detection by nitroblue tetrazolium (NBT, 1 mg mL^-1^) staining were performed according to the published methods (Jiang et al., 2012; Yang et al., 2014).

### Antioxidant Enzyme Assays

Seven-day-old seedlings were treated with 100 mM NaCl for 2 days, and then root samples were harvested. Enzyme extractions were performed at 4°C as described (Wang et al., 2016). Enzyme activities of superoxide dismutase (SOD) and catalase (CAT) were measured with SOD and CAT assay kits (Comin Biotechnology Co., Ltd., Suzhou, China) as described (Pan et al., 2016; Wang et al., 2016). Absorbances were measured using a spectrophotometer (DU730, Beckman Coulter) at 560 nm and 240 nm for SOD and CAT, respectively.

### Measurement of Na^+^ and K^+^ Content

Wild-type and two *iar4* mutant alleles were grown in a hydroponic system with half-strength Hoagland’s solution (Li et al., 2012) for 20 days, and then were treated with 100 mM NaCl for 4 days. Plant tissues (shoots and roots) were collected and dried for 3 days at 65°C. Dried samples were digested by HNO_3_ overnight at room temperature, and the digested solutions were further incubated in boiling water for 2 h. The samples were diluted with double-distilled water and measured for Na^+^ and K^+^ content by an absorption spectrometer (AA240Duo, Agilent, United States).

### Measurement of Zone Length, Cell Number, and Cell Length in MZ, EZ, and TZ

Three-day-old seedlings were transferred to MS medium supplemented with 100 mM NaCl or 200 μM GSH or both for 5 days. The roots were then placed in a mounting solution (80 g chloral hydrate, 10 mL glycerol, and 30 mL water) and were photographed with a Zeiss Axio Imager M2. The zone length, cell length, and cell number of meristem zone (MZ), transition zone (TZ), and elongation zone (EZ) in the cortex were measured and analyzed by ImageJ software according to the published methods (Verbelen et al., 2006; Jiang et al., 2016). At least 10 roots were used for each measurement, and the experiments were repeated at least three times.

### Fluorescence Detection

Four-day-old WT, *iar4-7*, and *iar4-8* seedlings expressing *ProPIN1:PIN1-GFP*, *ProPIN2:PIN2-GFP*, *ProPIN3:PIN3-GFP*, or *ProDR5:GFP* were transferred to MS medium or MS medium containing 100 mM NaCl or 200 μM GSH or both for 2 days. Root GFP fluorescence was detected with a confocal (Leica TCS SP8) microscope with excitation at 488 nm and emission at 525 nm. The relative fluorescence was analyzed with ImageJ software, and the fluorescence in WT on MS medium was set as 100%. At least 10 roots were used for each experiment, and at least three independent experiments were performed.

### Statistical Analysis

Differences among treatments or genotypes were assessed by Student’s *t*-test. The values were considered statistically significant at *P* < 0.05.

## Results

### *iar4* Mutants Were Hypersensitive to Salt Stress Conditions

**FIGURE 1:**
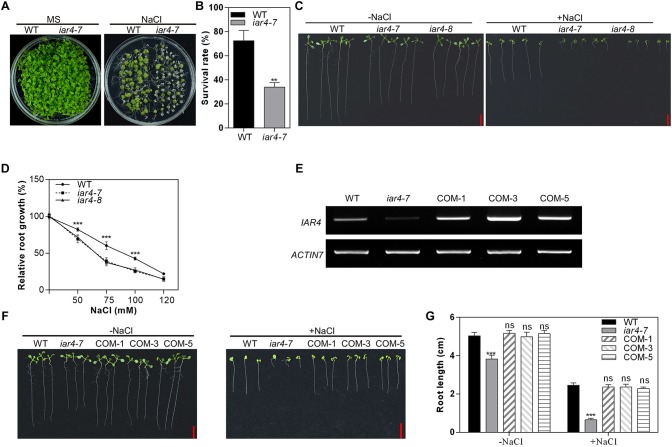
*iar4* mutants were hypersensitive to NaCl treatment. **(A)** The growth phenotype of wild-type (WT) and *iar4-7* on MS medium with or without 150 mM NaCl for 3 weeks. **(B)** The survival rates of the WT and *iar4-7* as described in **(A)**. ^∗∗^*P* < 0.01. **(C)** The root growth phenotype of *iar4-7*, *iar4-8*, and WT on MS medium containing 0 or 100 mM NaCl. **(D)** Relative root growth of *iar4-7*, *iar4-8*, and WT on MS medium containing various NaCl concentrations. Their real root length was in Supplementary Figure S2C. ^∗∗∗^*P* < 0.001. **(E)** The expression level of *IAR4* in WT, *iar4-7*, and *IAR4* complementation lines COM-1, COM-3, and COM-5. *ACTIN7* was used as a control. **(F,G)** The root length of WT and mutant plants grown on MS medium with or without 100 mM NaCl for 10 days. ^∗∗∗^*P* < 0.001. ns, not significant. Data presented were means ± SD, *n* = 3, each with 10 roots. Bars = 1 cm.

To identify the mutants that exhibit differential growth performance under salt stress conditions, we screened around 100 T-DNA insertion lines on MS medium plus 150 mM NaCl, in each of which one salt-inducible and auxin-responsive gene was disrupted. One mutant line SALK_091909 (referred to as *iar4-7*) (Steinwand et al., 2014) was screened out, as it was hypersensitive to NaCl treatment and had a significantly reduced survival rate than WT did (Figure 1A,B). However, there was no significant difference in growth performance between WT and *iar4-7* under normal growth conditions. *IAR4*, encoding a putative mitochondrial pyruvate dehydrogenase E1α-subunit (LeClere et al., 2004), was reported to regulate auxin homeostasis (Quint et al., 2009).

Salinity is also known to inhibit primary root growth. We next determined the primary root growth of the *iar4* mutants under salinity conditions. We obtained another T-DNA insertion allele of *iar4*, SALK_137286, here named as *iar4-8*. These two mutant alleles, *iar4-7* and *iar4-8*, were both determined as knock-down mutants by our genotyping analyses (Supplementary Figure S1). Three-day-old seedlings of WT, *iar4-7*, and *iar4-8* were transferred to MS medium plus 0 or 100 mM NaCl for 7 days (Figure 1C). The root length in *iar4-7* and *iar4-8* was slightly shorter than that of WT without NaCl treatment, consistent with a previous study (LeClere et al., 2004). NaCl treatment greatly inhibited the primary root growth of these genotypes, however, the inhibitions in the *iar4-7* and *iar4-8* seedlings were greatly exaggerated (Figure 1C and Supplementary Figures S2A,B). These results suggest that *IAR4* dysfunction leads to root growth retardation under salt stress conditions.

To better understand how the NaCl concentration range affects the primary root sensitivity, 3-day-old *iar4-7*, *iar4-8*, and WT seedlings were transferred to MS medium containing different NaCl concentrations. NaCl treatment inhibited primary root growth more in the *iar4* mutants than in the WT on MS medium supplemented with 75 mM or 100 mM NaCl, and led to the decreased relative root growth rate in the *iar4* mutants (Figure 1D). When the NaCl concentration was increased up to 120 mM or even greater, there were no significant differences in the relative root growth rate among *iar4-7*, *iar4-8*, and WT (Figure 1D and Supplementary Figure S2B). These findings suggest that 75 mM and 100 mM are suitable concentrations to distinguish the inhibition of primary root growth between the *iar4* mutants and WT. The 100 mM concentration was chosen for further experiments in the study.

To further confirm that the salt hypersensitive responses of the *iar4* mutants are due to the *IAR4* mutation, a genomic DNA fragment containing *IAR4* with its native promoter was introduced into one *iar4* allele, *iar4-7*, since two *iar4* mutant alleles used in this study showed similar phenotypes under salt stress conditions. The responses of three randomly selected *IAR4*-expressing *iar4-7* lines to salt stress conditions were tested (Figure 1E). As expected, *IAR4* expression not only restored the slightly short primary root phenotype of *iar4-7* on NaCl-free MS medium, but also complemented the NaCl-hypersensitive primary root growth phenotype (Figure 1F,G). These results demonstrate that *IAR4* is involved in the primary root growth under salt stress conditions.

### *iar4* Mutants Conferred Elevated Na^+^ Levels in Roots and Shoots

**FIGURE 2:**
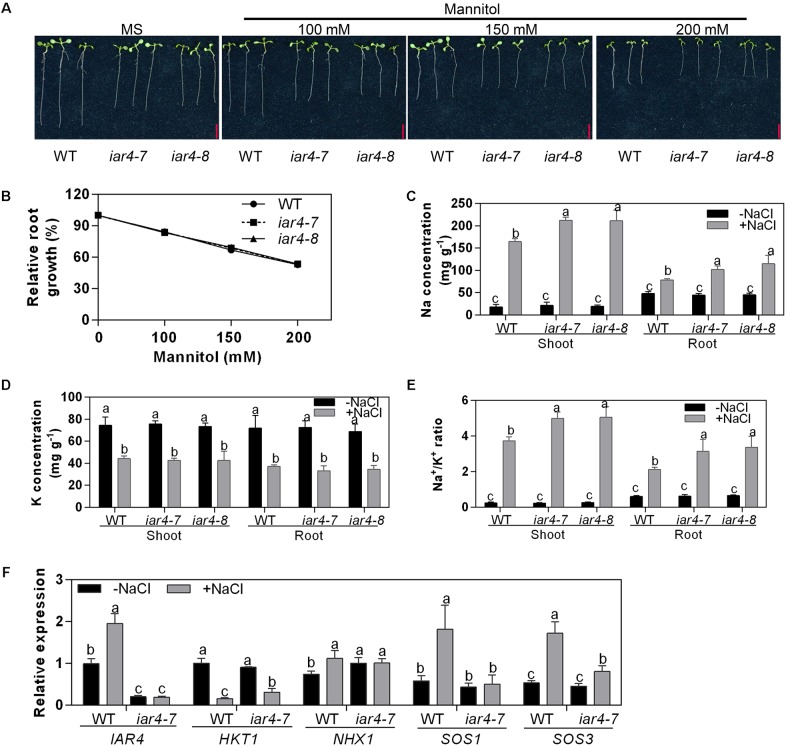
*iar4* mutants conferred elevated cytoplasm Na^+^ under salt stress conditions. **(A)** The root growth of *iar4-7*, *iar4-8*, and WT on MS medium supplemented with mannitol for 5 days. **(B)** Relative root growth of *iar4-7*, *iar4-8*, and WT in **(A)**, root length was expressed relative to those on MS medium. Data presented were means ± SD, *n* = 3, each with 10 roots. Bars = 1 cm. **(C,D)** Na^+^ and K^+^ contents in shoots and roots of 20-day-old plants grown in a hydroponic system treated with or without 100 mM NaCl for another 4 days. Data presented were means ± SD, *n* = 3. Different letters above the error bars indicate significant difference at *P* < 0.01 for **(C)** and at *P* < 0.05 for **(D)**. **(E)** Na^+^/K^+^ ratio in shoots and roots calculated from the data in **(C,D)**. Different letters above error bars indicate significant difference at *P* < 0.05. **(F)** Expression levels of *IAR4*, *HKT1*, *SOS1*, *SOS3*, and *NHX1* in roots treated with or without 100 mM NaCl for 8 h determined by qPCR. Data presented were means ± SD, *n* = 3. Different letters above error bars indicate significant difference at *P* < 0.05.

Salt stress is harmful to plants due to its osmotic stress or toxicity. To investigate which mechanism is responsible for the salt-hypersensitive primary root growth in the *iar4-7* and *iar4-8* mutants, 3-day-old seedlings were transferred to MS medium supplemented with mannitol for 5 days, which was considered as osmotic stress. There were no obvious differences in the relative root growth rate in the *iar4-7*, *iar4-8*, and WT under mannitol treatment (Figure 2A,B and Supplementary Figure S3). This suggests that the root hypersensitive response to salt stress in the *iar4* mutants may not be caused by osmotic stress.

Elevated salinity can cause overaccumulation of Na^+^ levels and reduction of K^+^ levels in cells (Shabala et al., 2007). We treated 20-day-old liquid-cultured *iar4* mutants and WT with 100 mM NaCl for 4 days, and measured Na^+^ and K^+^ content in shoots and roots. NaCl treatment increased the Na^+^ levels and decreased the K^+^ levels in both tissues in *iar4-7*, *iar4-8*, and WT (Figure 2C,D). However, *iar4* mutants accumulated greater levels of Na^+^ in both roots and shoots, and had similar K^+^ levels in both tissues as WT did (Figure 2D), which led to a greater Na^+^/K^+^ ratio in the *iar4-7* and *iar4-8* plants (Figure 2E). Without NaCl treatment, no significant differences were detected in Na^+^ and K^+^ content, and similar Na^+^/K^+^ ratios were observed in both tissues of *iar4* mutants and WT (Figure 2C). These results suggest that the phenotypes of primary root retardation and low survival rate in the *iar4* mutants under salt stress conditions may be likely due to Na^+^ overaccumulation and high Na^+^/K^+^ ratio (Figure 1).

In Arabidopsis, Na^+^ homeostasis is coordinated by Na^+^ transporters. HKT1 is known to uptake Na^+^ into root cells, Na^+^/H^+^ antiporter SOS1 exports Na^+^ out of the cell, and NHX1 transports Na^+^ into the vacuole for sequestration (Shi et al., 2000; Apse et al., 2003; Platten et al., 2006). To elucidate whether these transporters are involved in Na^+^ over-accumulation in the *iar4* mutant plants, the expression levels of *HKT1*, *SOS1*, and *NHX1* in roots were detected. NaCl treatment inhibited the expression of *HKT1* in both genotypes, but *HKT1* expression was slightly higher in *iar4-7* than in WT. The expression levels of *NHX1* were comparable in WT and *iar4-7* with and without NaCl treatment. Surprisingly, *SOS1* was greatly induced by NaCl treatment in WT, however, its induction in *iar4-7* was totally blocked (Figure 2F). These results suggest that the overaccumulation of Na^+^ in the *iar4* mutants may be mainly caused by the failure to exclude Na^+^ out of the root cells. Activation of the SOS signaling pathway has long been recognized as a key mechanism for Na^+^ exclusion and ion homeostasis control at the cellular level (Zhu, 2000). We then checked the expression level of *SOS3*, which is an upstream regulator of *SOS1* and perceives the increase of cytosolic Ca^2+^ triggered by excess Na^+^. Interestingly, we found the induction of *SOS3* by NaCl treatment was also strongly impaired in the *iar4* mutant (Figure 2F). These results indicate that increased Na^+^ content in *iar4* is mainly caused by less Na^+^ exclusion out of root cells by the SOS1 transporter, probably through the SOS signaling pathway.

### *iar4* Mutants Accumulated More ROS in Root Tips Under Salt Stress Conditions

**FIGURE 3:**
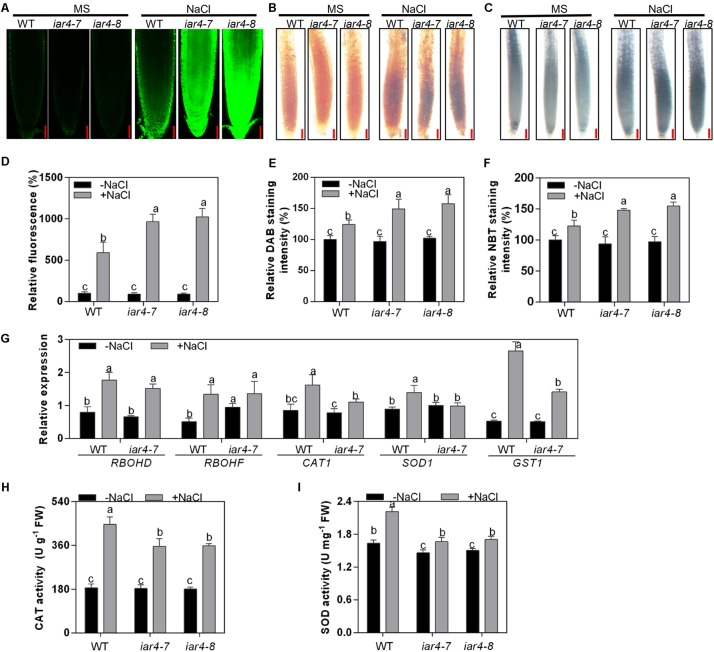
*iar4* mutants accumulated more ROS under salt treatment. **(A)** H_2_DCFDA and **(B)** DAB staining for H_2_O_2_ in the primary root of 5-day-old *iar4-7*, *iar4-8*, and WT with or without NaCl treatment. Bars = 50 μm. **(C)** NBT staining for superoxide in the primary root of *iar4-7*, *iar4-8*, and WT with or without NaCl treatment. Bars = 50 μm. **(D)** The fluorescence intensity, **(E)** DAB staining intensity and **(F)** NBT staining intensity in *iar4-7*, *iar4-8*, and WT with or without NaCl treatment was determined by ImageJ in **(A–C)**, respectively. The intensity in WT without NaCl treatment was set as 100%. In **(D–F)**, data presented were means ± SD, *n* = 3, each with 10 roots. Different letters above error bars indicate significant difference at *P* < 0.001. **(G)** Quantitative PCR analyses of ROS production genes (*RBOHD*, *RBOHF*) and ROS scavenging genes (*CAT1*, *SOD1*, *GST1*) in roots treated with or without NaCl for 8 h. **(H,I)** Activities of catalase (CAT) and superoxide dismutase (SOD) in roots of *iar4-7*, *iar4-8*, and WT with or without NaCl treatment for 2 days. Data presented were means ± SD, *n* = 3. –NaCl, without NaCl; +NaCl, with 100 mM NaCl. Different letters above error bars indicate significant difference at *P* < 0.05.

Salt stress induces ROS accumulation. To determine whether the salt-induced root inhibition in the *iar4* mutants is via ROS homeostasis pathway, we compared the levels of H_2_O_2_ (by H_2_DCF-DA and DAB staining) and superoxide (by NBT staining) in roots. Without NaCl treatment, *iar4-7*, *iar4-8*, and WT roots showed similar levels of H_2_O_2_ and superoxide. NaCl treatment induced H_2_O_2_ and superoxide levels in the *iar4* mutants and WT, but the inductions were much greater in the *iar4* mutants (Figure 3A–F). These results reveal that *IAR4* dysfunction increases ROS levels in roots under salt stress conditions.

Reactive oxygen species homeostasis is balanced by ROS production and scavenging. To understand whether the greater ROS level in the *iar4* mutants is due to the defect of ROS production or scavenging, the expression patterns of major ROS production genes (*RBOHD* and *RBOHF*) and ROS scavenging genes (*CAT1*, *SOD1*, and *GST1*) were determined in the roots of 7-day-old *iar4-7* and WT seedlings treated with 100 mM NaCl for 8 h. NaCl treatment induced the expression of *RBOHD* and *RBOHF* in *iar4-7* and WT, and their inductions were comparable. The expression levels of *CAT1*, *SOD1*, and *GST1* were similar between *iar4-7* and WT on MS medium, however, their inductions by NaCl treatment were less in the *iar4-7* mutant compared with those in WT (Figure 3G). These results illustrate that the increased ROS accumulation in the *iar4* mutants is mainly caused by the reduced ROS scavenging, rather than ROS production. To further prove this, the activities of two key ROS-scavenging enzymes, CAT, which scavenges H_2_O_2_, and SOD, which scavenges superoxide, were measured in the roots of *iar4-7*, *iar4-8*, and WT with or without NaCl treatment. NaCl treatment increased the activities of CAT and SOD in these genotypes, however, CAT and SOD activities were much less in *iar4-7* and *iar4-8* than in WT (Figure 3H,I), consistent with the expression levels of ROS scavenging genes (Figure 3G). Taken together, our results suggest that the primary root growth retardation in the *iar4* mutants under salt stress conditions is likely due to the oxidative status caused by ROS accumulation.

### Root Meristem Activity Was Impaired in the *iar4* Mutants Under Salt Stress Conditions

**FIGURE 4:**
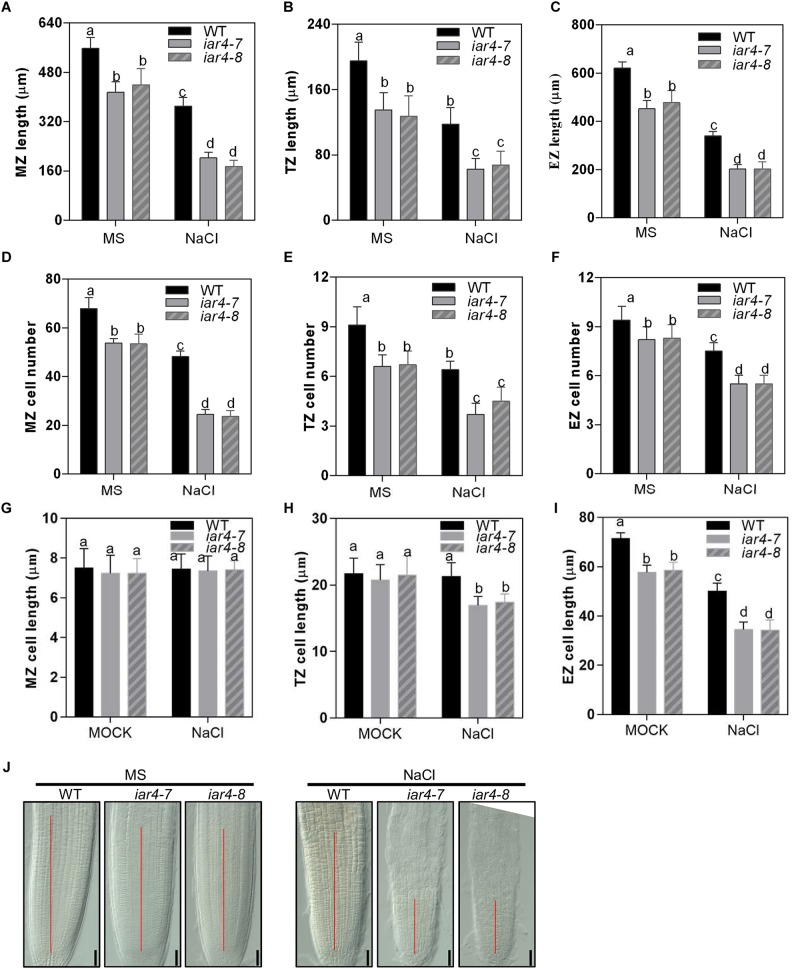
Root meristem activity in the *iar4* mutants was reduced under NaCl treatment. **(A–C)** The MZ, TZ, and EZ lengths of *iar4-7*, *iar4-8*, and WT with 0 or 100 mM NaCl treatment. Different letters above error bars indicate significant difference at *P* < 0.001 for **(A,C)**, at *P* < 0.01 for **(B)**. **(D–F)** The corresponding cell number in the MZ, TZ, and EZ with 0 or 100 mM NaCl treatment. Different letters above error bars indicate significant difference at *P* < 0.01. **(G–I)** The cell length of the MZ, TZ, and EZ with or without NaCl treatment. MZ, meristem zone, TZ, transition zone, EZ, elongation zone. Different letters above error bars indicate significant difference at *P* < 0.01 for **(H)**, at *P* < 0.05 for **(I)**. **(J)** The root meristem size in *iar4-7*, *iar4-8*, and WT with or without NaCl treatment. Data presented were means ± SD, *n* = 3, each with 10 roots.

To detect how NaCl or NaCl-induced ROS affects root length, we measured root zone length, cell number, and cell length of MZ, TZ, and EZ in *iar4-7*, *iar4-8*, and WT roots with or without NaCl treatment. Without NaCl treatment *iar4-7* and *iar4-8* exhibited less MZ length and fewer MZ cell numbers than WT did. NaCl treatment reduced the MZ length and cell number more in the *iar4* mutants than in WT, but the MZ cell length was comparable in WT and *iar4* roots with or without NaCl treatment (Figure 4A,D,F). The TZ was shorter and had fewer cell numbers in the *iar4* mutants than in WT under both conditions. Although the TZ cell length was similar in the *iar4* mutants and WT without NaCl treatment, NaCl treatment reduced the TZ cell length more in the *iar4* mutants than in WT (Figure 4B,E,H). Without NaCl treatment the EZ length was also shorter in the *iar4* mutants, with fewer cell numbers and shorter cell lengths. NaCl treatment reduced the EZ cell length and cell number more in the *iar4* mutants than in WT, leading to further inhibited EZ length in the *iar4* mutants (Figure 4C,F,I). These results indicate that the *iar4* mutation reduces root meristem activity and increases the sensitivity of all three root zones to NaCl treatment, by reducing the cell number in all three zones, as well as the cell elongation in the TZ and EZ. To further reveal that the reduced cell number in the *iar4* mutants is caused by the reduced cell division activity, the expression levels of cell cycle marker genes *CYCB1;1* (AT4G37490) and *CYCB1;2* (AT5G06150) (Weimer et al., 2016; Zhang et al., 2018) were detected. The expression levels of *CYCB1;1* and *CYCB1;2* were significantly less in the *iar4* mutants than in WT without salt stress, and their repressions by salt stress were enhanced in the *iar4* mutants (Supplementary Figure S4). These results were consistent with the cell number and root length phenotypes with or without NaCl treatment (Figure 4). These results suggest that the cell division activity in the *iar4* mutant is reduced under normal and salt stress conditions.

### GSH Treatment Partially Alleviated the Salt-Hypersensitive Root Growth Phenotype in the *iar4* Mutants

**FIGURE 5:**
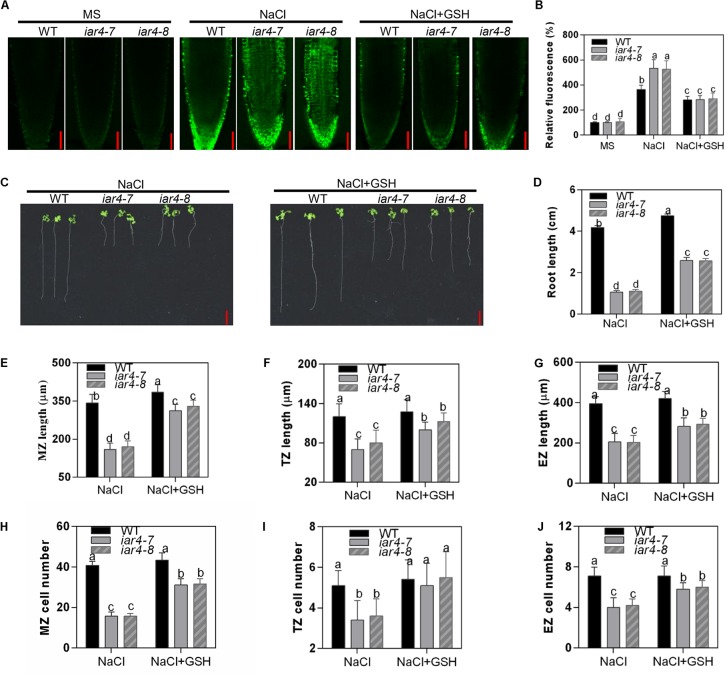
GSH treatment partially rescued the salt-hypersensitive root growth phenotype of *iar4* mutants. **(A)** H_2_DCFDA staining for H_2_O_2_ in the primary root of 5-day-old *iar4-7*, *iar4-8*, and WT with 100 mM NaCl or 100 mM NaCl plus 200 μM GSH. Bars = 50 μm. **(B)** The fluorescence intensity in *iar4-7*, *iar4-8*, and WT was determined by ImageJ in **(A)**. The intensity in WT without NaCl treatment was taken as 100%. **(C)** The primary root growth of *iar4-7*, *iar4-8*, and WT plants with NaCl or NaCl + GSH. **(D)** Relative root growth was statistically analyzed as shown in **(A)**. The root length is expressed relative to that of WT with NaCl treatment. Different letters above error bars indicate significant difference at *P* < 0.01. Bars = 1 cm. **(E–G)** The MZ, TZ, and EZ length of *iar4-7*, *iar4-8*, and WT seedlings with NaCl, with or without GSH. Different letters above error bars indicate significant difference at *P* < 0.05. **(H–J)** The corresponding cell number of MZ, TZ, and EZ treated with NaCl or NaCl + GSH. MZ, meristem zone, TZ, transition zone, EZ, elongation zone. Different letters above error bars indicate significant difference at *P* < 0.01 for **(J)**, at *P* < 0.001 for **(H,I)**. Data presented were means ± SD, *n* = 3, each with 10 roots measured.

H_2_O_2_ is a major ROS species in plants under abiotic stresses (Krishnamurthy and Rathinasabapathi, 2013), and can be scavenged by GSH antioxidant (Yang et al., 2014). To determine if the salt-inhibition of root growth in the *iar4* mutants is caused by ROS overaccumulation, we investigated whether supplement of GSH could restore the root phenotype of the *iar4* mutants under salt stress conditions. Supplement of 200 μM GSH greatly reduced ROS levels in WT and *iar4* mutants that were promoted by NaCl treatment (Figure 5A,B and Supplementary Figures S5A,B), suggesting GSH (200 μM) is functional to scavenge ROS in the roots. We next detected the effect of GSH treatment on the primary root growth performance in the *iar4* mutants under salt stress. NaCl treatment significantly inhibited the root length in the *iar4* mutants, while GSH treatment partially restored the salt-hypersensitive root growth in the *iar4* mutants (Figure 5C,D). These findings suggest that reducing ROS level can partially alleviate the root growth retardation in the *iar4* mutants under salt stress.

We were also interested to know whether GSH treatment could restore cell division and cell elongation in the *iar4* mutants, which were inhibited by accumulated ROS. For better interpretation of this, we firstly tested the effect of exogenous H_2_O_2_ treatment with or without GSH supplement on cell division and cell elongation in WT roots. Exogenous H_2_O_2_ (0.5 mM) suppressed the cell number and cell length in WT, and 200 μM GSH treatment could restore the phenotypes (Supplementary Figures S5C–H). We then treated 3-day-old WT, *iar4-7*, and *iar4-8* seedlings with 200 mM GSH plus NaCl for 5 days, and determined the zone length and cell number in the MZ, TZ, and EZ. GSH treatment not only greatly rescued the MZ, TZ, and EZ lengths in *iar4-7* and *iar4-8*, which were suppressed by NaCl (Figure 5E–G and Supplementary Figures S5I,J), but also greatly rescued the cell numbers in each zone (Figure 5H–J). These results suggest that GSH treatment reduces the oxidation status (ROS) in *iar4* root tips, which then decreases the salt inhibition of root growth.

### Salt Stress Suppressed the Expression Levels of *ProDR5:GFP* and Auxin Carriers More in *iar4* Root Tips, Which Could Be Largely Reversed by GSH

**FIGURE 6:**
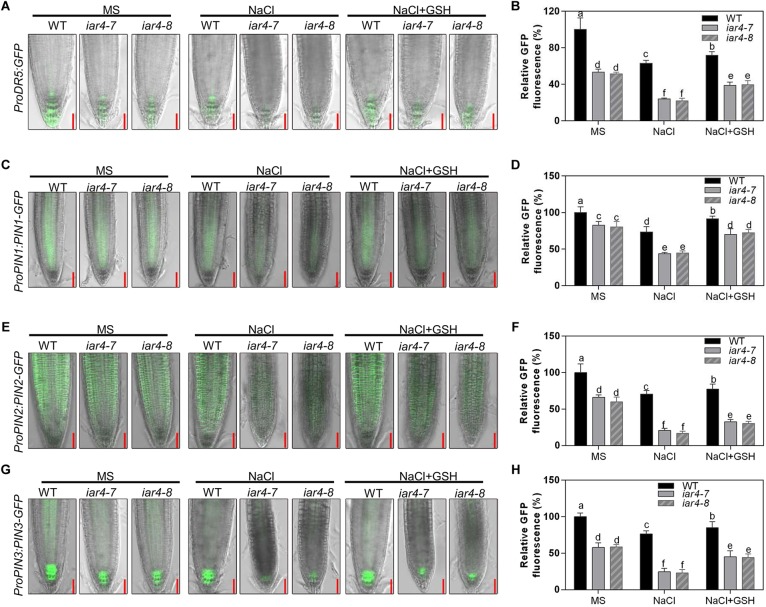
GSH treatment partially reversed the expression levels of *ProDR5:GFP*, *ProPIN1:PIN1-GFP*, *ProPIN2:PIN2-GFP* and *ProPIN3:PIN3-GFP*, which were suppressed by NaCl treatment. **(A)** The expression of *ProDR5:GFP* under different treatments. **(B)** The fluorescence of *ProDR5:GFP* in **(A)**. Fluorescence is expressed relative to that of the WT on MS medium without treatment. Four-day-old seedlings were transferred to MS medium containing NaCl, GSH, or NaCl plus GSH for 2.5 days. Bars = 50 μm. Different letters above error bars indicate significant difference at *P* < 0.05. **(C)** *ProPIN1:PIN1-GFP* expression under different treatments. **(D)** The fluorescence of *ProPIN1:PIN1-GFP* in **(C)**. Fluorescence is expressed relative to that of WT on MS medium without treatment. Four-day-old seedlings were transferred to MS medium containing NaCl, GSH, or NaCl plus GSH for 2 days. Bars = 50 μm. **(E)** *ProPIN2:PIN2-GFP* expression under different treatments. **(F)** The fluorescence of *ProPIN2:PIN2-GFP* in **(E)**. Four-day-old seedlings were transferred to MS medium containing NaCl, GSH, or NaCl plus GSH for 2 days. Bars = 50 μm. **(G)** *ProPIN3:PIN3-GFP* expression under different treatments. **(H)** The fluorescence of *ProPIN3:PIN3-GFP* in **(G)**. Four-day-old seedlings were transferred to MS medium containing NaCl, GSH, or NaCl plus GSH for 2 days. Bars = 50 μm. Fluorescence is expressed relative to that of WT on MS medium without treatment. Different letters above error bars indicate significant difference at *P* < 0.01. Data presented in **(B,D,F,H)** were means ± SD, *n* = 3, each repeat with 10 roots. Different letters above error bars indicate significant difference at *P* < 0.05.

Auxin is a major hormone that regulates root growth. Previous studies have shown that *iar4* mutation exhibits increased IAA-Glu and similar endogenous auxin levels as Col-0. Supplement of IAA can complement the *iar4* mutant phenotypes (LeClere et al., 2004; Quint et al., 2009), suggesting that IAR4 is involved in auxin homeostasis rather than in auxin biosynthesis. To see whether the reduced root growth under salt stress treatment in the *iar4* mutants is due to an impairment of auxin level in root tips and responses, we crossed *iar4-7* and *iar4-8* with an auxin-responsive marker line *ProDR5:GFP*, which reports auxin accumulation and distribution in root tips (Friml et al., 2003). NaCl treatment decreased *ProDR5:GFP* expression in the *iar4* mutants and WT, consistent with previous reports that salt stress inhibited auxin biosynthesis or auxin distribution (Jiang et al., 2016). However, the *ProDR5:GFP* expression in the *iar4* root tips was much inhibited (Figure 6A,B). Note that without NaCl treatment, *ProDR5:GFP* expression in the *iar4* root tips was also less than that in WT (Figure 6A,B), in agreement with the previous study (Quint et al., 2009).

*iar4* mutants overaccumulated ROS and exhibited reduced auxin distribution in root tips. We next tested the effect of GSH on *ProDR5:GFP* expression in the *iar4* mutants and WT under salt stress conditions. GSH treatment slightly increased *ProDR5:GFP* expression in both WT and *iar4* root tips compared with controls on MS medium. Under NaCl conditions, GSH treatment increased *ProDR5:GFP* expression more in the *iar4* mutants than in WT, which then diminished the differences in primary root length between the *iar4* mutants and WT (Figure 6A,B). These results demonstrate that the less expression of *ProDR5:GFP* in the *iar4* mutants under salt stress conditions is likely due to the high oxidation status in root tips.

Since auxin transporters play crucial roles in modulating root growth, we then detected *ProPIN1:PIN1-GFP*, *ProPIN2:PIN2-GFP*, and *ProPIN3:PIN3-GFP* expression levels in the *iar4* mutants and WT. Their expression levels were less in the *iar4* mutants than in WT without NaCl treatment (Figure 6C–H), which contributed to the reduced expression of *ProDR5:GFP* in the *iar4* mutants (Figure 6A,B), consistent with the previous study (Quint et al., 2009). NaCl treatment suppressed the expression levels of *ProPIN1:PIN1-GFP*, *ProPIN2:PIN2-GFP*, and *ProPIN3:PIN3-GFP* in the *iar4* mutants and WT, but their suppressions were dramatically exaggerated in the *iar4* mutants compared with those in WT (Figure 6C–H). GSH treatment greatly diminished NaCl-inhibition of *ProPIN1:PIN1-GFP*, *ProPIN2:PIN2-GFP*, and *ProPIN3:PIN3-GFP* expression levels in the *iar4* mutants. Auxin homeostasis depends on auxin uptake and efflux. We next detected *AUX1* expression under salt stress conditions with or without GSH treatment. The expression of *AUX1* in the *iar4* mutant was much inhibited by salt stress, and could also be restored by GSH treatment (Supplementary Figure S6), suggesting the reduced auxin uptake also contributes to the disturbed auxin homeostasis in the *iar4* mutants. Together with the previous study which shows that *iar4* mutation does not change the content of free IAAs in roots, shoots, or whole plants (Quint et al., 2009), our results indicate that the reduced expression of *ProDR5:GFP* and the reduced auxin carriers in *iar4* roots under salt stress conditions are likely due to the elevated ROS level. These results also suggest ROS accumulated by salt stress reduces the auxin distribution in *iar4* roots, and that ROS acts as a vital mediator between salt and auxin distribution in roots.

### Exogenous Auxin Largely Recovered the Salt-Hypersensitive Root Growth Phenotype of *iar4*

**FIGURE 7:**
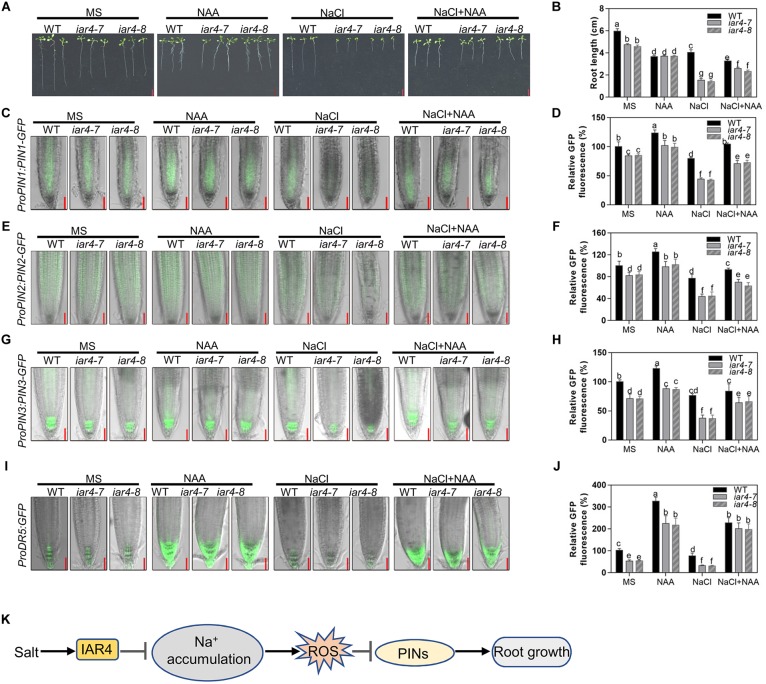
Exogenous auxin greatly restored the salt-inhibited root growth of *iar4* mutants. **(A)** The root growth phenotype of WT, *iar4-7* and *iar4-8* treated with 100 mM NaCl or 100 mM NaCl plus 0.1 μM NAA. **(B)** The root length was statistically analyzed as shown in **(A)**. Data presented were means ± SD, *n* = 3, each repeat with 10 roots. Different letters above error bars indicate significant difference at *P* < 0.01. Bars = 1 cm. **(C)** *ProPIN1:PIN1-GFP* expression under different treatment. **(D)** The fluorescence of *ProPIN1:PIN1-GFP* in **(C)**. Fluorescence is expressed relative to that of WT on MS medium without treatment. **(E)** *ProPIN2:PIN2-GFP* expression under different treatment. **(F)** The fluorescence of *ProPIN2:PIN2-GFP* in **(E)**. Fluorescence is expressed relative to that of WT on MS medium without treatment. **(G)** *ProPIN3:PIN3-GFP* expression under different treatment. **(H)** The fluorescence of *ProPIN3:PIN3-GFP* in **(G)**. **(I)** *ProDR5:GFP* expression under NAA or NaCl or NAA plus NaCl treatment. Bars = 50 μm. **(J)** The fluorescence of *ProDR5:GFP* in **(I)**. Fluorescence is expressed relative to that of WT without treatment. Data presented were means ± SD, *n* = 3, each repeat with 10 roots. Different letters above error bars indicate significant difference at *P* < 0.01. **(K)** A proposed model for IAR4 in root growth under salt stress conditions. Salt stress induces *IAR4* expression to negatively regulate NaCl accumulation. NaCl accumulation promotes ROS production and ROS negatively controls root growth by regulating PIN-mediated auxin polar transport.

The reduced *DR5-GFP* expression and root meristem activity in the *iar4* mutants may be the main reason for the short primary root growth under salt stress. Reducing the ROS level in the *iar4* mutants could reverse this phenotype. We performed experiments to see whether supplement of auxin could also recover the salt inhibition of root growth in the *iar4* mutants. WT and *iar4* mutant plants were grown on MS medium containing 0.1 μM NAA in the presence or absence of 100 mM NaCl. Without NaCl treatment, supplement of NAA inhibited the primary root growth of both WT and *iar4* mutants, but the inhibition in the *iar4* mutants was reduced, which resulted in the similar root lengths in the WT and *iar4* mutants (Figure 7A,B and Supplementary Figure S7). With NaCl treatment, supplement of NAA further inhibited the primary root growth of WT compared with that grown on MS medium plus NaCl or NAA. In contrast, NAA largely abolished the inhibition of primary root length by NaCl treatment in the *iar4* mutants (Figure 7A,B). Similar effects were observed in IAA treatment (Supplementary Figure S8).

We also detected the expression levels of *ProDR5:GFP*, *ProPIN1:PIN1-GFP*, *ProPIN2:PIN2-GFP*, and *ProPIN3:PIN3-GFP* in WT and *iar4* root tips under these conditions. NAA treatment together with NaCl resulted in the greater expression of *ProDR5:GFP*, *ProPIN1:PIN1-GFP*, *ProPIN2:PIN2-GFP*, and *ProPIN3:PIN3-GFP* in the *iar4* mutants and WT (Figure 7C–H). However, the increase was greater in the *iar4* mutants than that in WT, which led to only the slight differences in primary root length between them (Figure 7A,B). These data demonstrate that auxin treatment can largely restore the salt inhibition of root growth in *iar4*, suggesting exogenous auxin compensates the NaCl-inhibited auxin accumulation in *iar4* root tips.

## Discussion

Salinity is a major abiotic stress that seriously affects crop production (Munns and Tester, 2008). In addition, the increasing soil salinity is threatening the sustainability of food production. The root is the direct and first organ to sense and respond to the increasing salinity, however, the underlying mechanisms still remain largely unknown. The present study demonstrates that IAR4 is essential for primary root growth under salt stress, by integrating the ROS and auxin pathways. We found that *iar4* mutants showed a strong hypersensitive phenotype to salt stress. We further found that *iar4* mutants accumulated elevated Na^+^ and ROS levels. The auxin distribution in root tips was much less in the *iar4* mutants under salt stress, because of the reduced expression of auxin response reporter and auxin carriers, which led to the reduced root meristem activity and root growth. GSH treatment could partially recover the reduced root meristem activity and root growth retardation in the *iar4* mutants induced by salt stress conditions.

Diverse mechanisms are adopted by plants in salt tolerance regulation, including ionic toxicity, osmotic stress, and oxidative stress on plant cells (Zhu, 2002; Zhu et al., 2007). Our results showed that the hypersensitive primary root growth in the *iar4* mutants was mainly due to the Na^+^ toxicity and oxidative stress, rather than the osmotic effect. There were several reasons. First, osmotic stress by mannitol treatment had a similar effect on primary root growth in both WT and *iar4* mutant alleles (Figure 2A,B and Supplementary Figure S3). Second, *iar4* mutants accumulated much greater Na^+^ levels and exhibited a greater Na^+^/K^+^ ratio in both roots and shoots under salt stress conditions (Figure 2), which were contributed by significantly reduced *SOS1* and *SOS3* expression. This is in agreement with a previous study that *sos1* mutation caused a salt hypersensitive phenotype (Shi et al., 2000). Third, ROS overaccumulated in the *iar4* mutants, due to the reduced ROS scavenging. Supplement of GSH greatly recovered the root growth retardation in the *iar4* mutants by NaCl treatment (Figure 3, 5 and Supplementary Figure S5). It was reported that ROS detoxification genes *ENH1* and *FeSOD* were directly regulated by SOS1 (Katiyar-Agarwal et al., 2006). Finally, the accumulated Na^+^ and ROS in the *iar4* mutants caused the reduction of root meristem activity, cell division, cell length, and root length (Figure 4). GSH treatment partially restored these phenotypes (Figure 5 and Supplementary Figure S5). These results demonstrate that the primary root growth retardation in the *iar4* mutants under salt stress is directly linked to oxidative stress (ROS).

Auxin is reported to be a vital regulator in root meristem maintenance (Swarup et al., 2002; Overvoorde et al., 2010), and its distributions and levels in root tips directly regulate root growth. IAR4 encodes a putative mitochondrial pyruvate dehydrogenase E1α-subunit. However, its function in linking glycolysis to the Krebs cycle is questionable, because Krebs cycle intermediates fail to rescue the *iar4* root phenotypes. IAA treatment could rescue the root phenotypes under normal growth conditions, suggesting IAR4 might specifically function in the auxin pathway (LeClere et al., 2004; Quint et al., 2009). Further experiments showed that free IAA levels in roots or shoots in the *iar4* mutants were comparable with those in Col-0, and IAA-Glu level was increased in the *iar4* mutants, suggesting that IAR4 might be involved in auxin homeostasis. In our study, *ProDR5:GFP* expression in *iar4* root tips was slightly reduced under normal growth conditions, consistent with the slightly shorter roots reported here (Figure 1, 2, 5, 7) and in previous studies (LeClere et al., 2004; Quint et al., 2009). Under salt stress, *ProDR5:GFP* expression was significantly decreased in the *iar4* mutants (Figure 6A,B), leading to strongly inhibited primary root growth. The heavily suppressed expression of auxin carriers (*ProPIN1:PIN1-GFP*, *ProPIN2:PIN2-GFP*, and *ProPIN3:PIN3-GFP*) in the *iar4* mutants under NaCl treatment suggested that the reduced auxin level in root tips was due to the reduced auxin transport. The greater auxin levels in the EZ and TZ further inhibited cell elongation in these zones and root length. When auxin (IAA or NAA) was supplemented, the expression levels of *ProPIN1:PIN1-GFP*, *ProPIN2:PIN2-GFP*, *ProPIN3:PIN3-GFP*, and *ProDR5:GFP* were recovered. These findings suggest that *IAR4* may mainly affect auxin distribution in roots under salt stress. IAR4 maintains the balance of free IAA and IAA-conjugates, and IAR4 is induced by salt treatment (Figure 2F). Under salt treatment, IAA-conjugates may be accumulated, and then free IAA is reduced in the *iar4* mutant. In this sense, the strongly inhibited primary root growth may also be caused by disturbed auxin homeostasis.

Salt stress induces ROS accumulation and suppresses auxin biosynthesis, homeostasis, and responses, which might be a strategy that plants use to tolerate salinity. However, how salt signals and root development are integrated by ROS-auxin crosstalk is still uncertain. Some reports show that ROS inhibits auxin biosynthesis and homeostasis (Nakagami et al., 2006; De Tullio et al., 2010; Blomster et al., 2011). Other reports show that auxin regulates ROS homeostasis (Joo et al., 2001; Schopfer et al., 2002; Joo et al., 2005; Peer et al., 2013). Under salinity stress conditions, *iar4* mutants accumulated more ROS (Figure 3) and exhibited reduced auxin levels in root tips than WT did (Figure 6, 7). Supplement of GSH or auxin partially restored the decreased expression of *ProDR5:GFP*, *ProPIN1:PIN1-GFP*, *ProPIN2:PIN2-GFP*, and *ProPIN3:PIN3-GFP* under salt stress conditions (Figure 6, 7), and greatly rescued the primary root growth retardation phenotype in the *iar4* mutants (Figure 5 and Supplementary Figure S5). These results suggest that reducing ROS levels in the *iar4* mutants can release the salt-inhibition of auxin distribution and homeostasis (Figure 6). These results demonstrate that the dramatically reduced auxin transport and the changed homeostasis in the *iar4* mutants are caused by higher ROS levels. It is also reported that SOS3 is required for auxin biosynthesis and polar auxin transport to root tips under salt stress conditions (Zhao et al., 2011). In the *iar4* mutants, salt-induction of *SOS3* was strongly inhibited (Figure 2F), indicating *IAR4* mediates primary root growth under salt stress via ROS-mediated regulation of auxin distribution, probably through a SOS-controlled pathway. SOS3 may be inhibited by metabolites, since IAR4 encodes a PDH E1α subunit, or be inhibited by auxin or auxin-conjugates, or other mechanisms. However, further experiments are needed to investigate.

## Conclusion

In conclusion, the present study identifies IAR4 that acts as a key player in primary root growth modulation under salt stress conditions. Furthermore, characterization of the roles of ROS and auxin in salt stress-regulated primary root growth leads to a model that IAR4 modulates primary root growth under salt stress conditions, mainly through ROS-mediated modulation of auxin distribution (Figure 7I). Under salt stress, *IAR4* is induced to prevent Na^+^ overaccumulation, probably through a SOS signaling pathway. Na^+^ accumulation promotes ROS production, which negatively modulates root growth by regulating PIN-mediated auxin polar transport in roots.

## Author Contributions

HH and YF planned and designed the research, analyzed the data, and wrote the manuscript. YF, YY, SC, and NN performed the experiments.

## Conflict of Interest Statement

The authors declare that the research was conducted in the absence of any commercial or financial relationships that could be construed as a potential conflict of interest.
