# Supplementary material for: Arabidopsis IAR4 Modulates Primary Root Growth Under Salt Stress Through ROS-Mediated Modulation of Auxin Distribution
Source: Front Plant Sci. 2019 Apr 25;10:522. doi: 10.3389/fpls.2019.00522 (PMC6494962; doi:10.3389/fpls.2019.00522)
Supplement: Supplementary file 1 [file Data_Sheet_1.doc]

**Supplementary Figures S1-S8 and Table S1**

The following Supporting Information is available for this article.

**Figure S1 |** Characterization of *iar4-7* and *iar4-8* T-DNA insertion mutants.

**Figure S2 |** Primary root growth under salt stress conditions.

**Figure S3 |** Primary root growth under mannitol stress conditions.

**Figure S4 |** *iar4* mutation reduced the cell division activity under salt stress conditions.

**Figure S5 |** GSH treatment partially reduced ROS levels and recovered the root length of *iar4* mutants under salt stress conditions.

**Figure S6 |** *AUX1* expressions in *iar4* and WT under NaCl stress conditions with and without GSH treatment.

**Figure S7 |** Exogenous NAA largely restored the salt-inhibited root growth of *iar4* mutants.

**Figure S8 |** Exogenous IAA largely restored the salt-inhibited root growth of *iar4* mutants.

**Table S1 |** Primers used in this study.


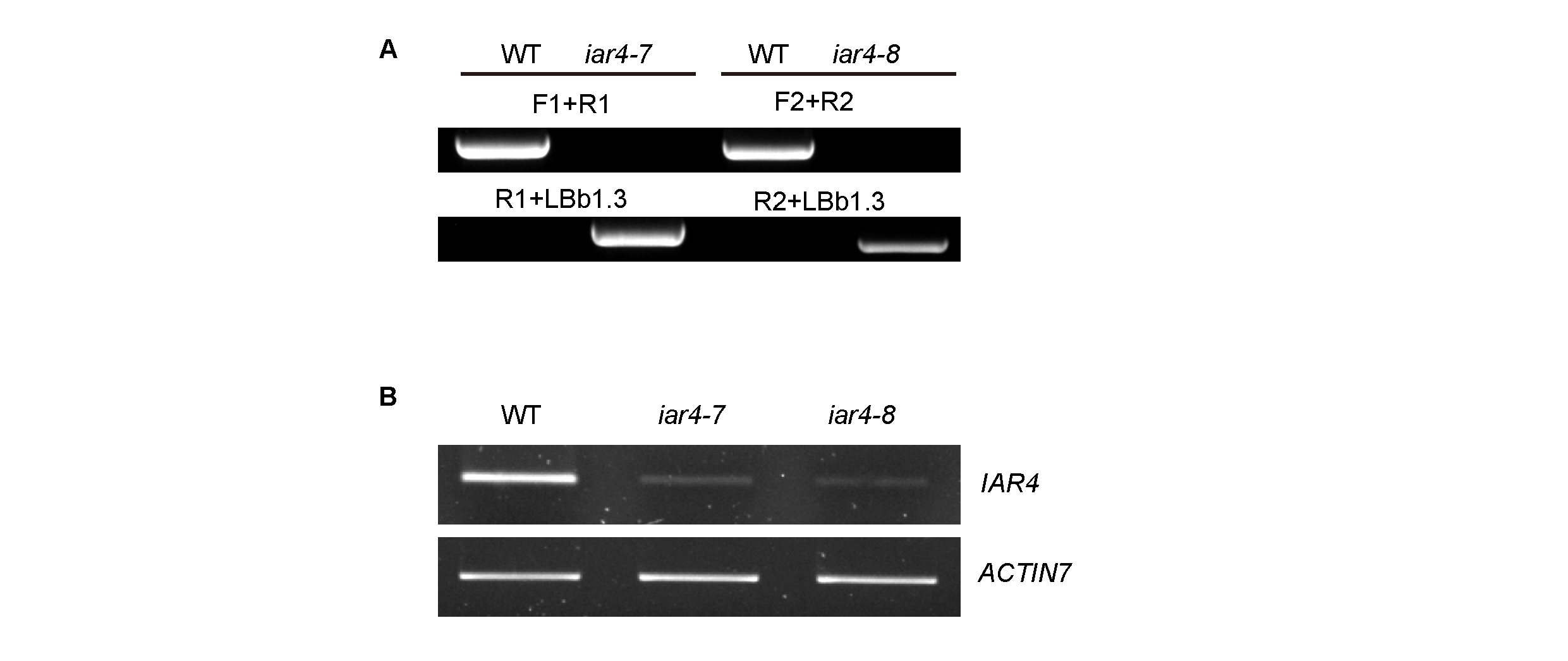


**Figure S1 |** Characterization of *iar4-7* and *iar4-8* T-DNA insertion mutants. **(A)** Genotyping of homozygous lines of *iar4-7* and *iar4-8*. LBb1.3 was the left border primer of the T-DNA insertion. **(B)** Expression of *IAR4* in *iar4-7* and *iar4-8* seedlings. *Actin7* was used as an internal control and was amplified for 26 circles. *IAR4* were amplified for 29 circles.


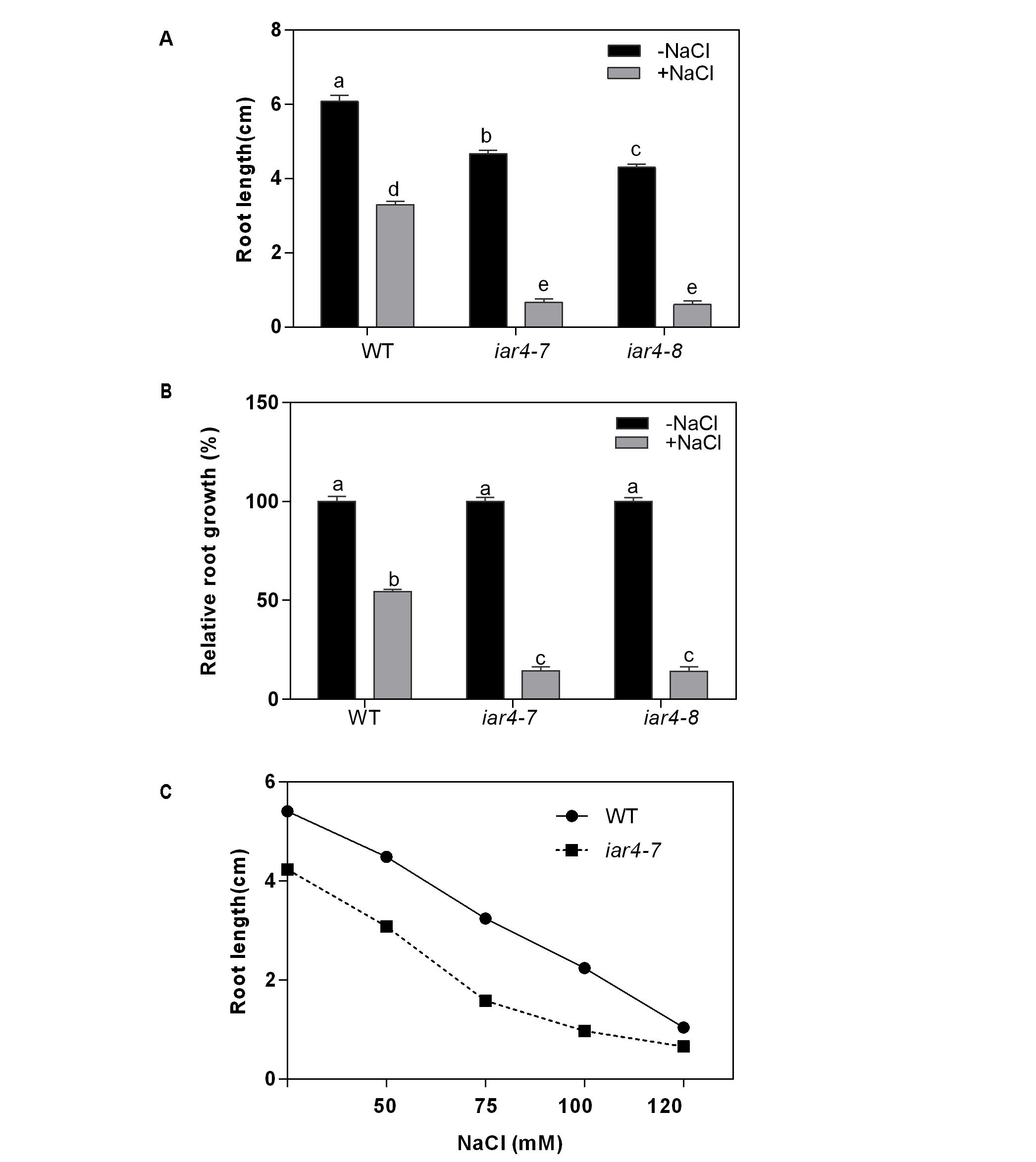


**Figure S2 |** Primary root growth under salt stress conditions. **(A)** Three-day-old WT, *iar4-7* and *iar4-8* plants were transferred to MS medium containing 0 or 100 mM NaCl for 7 days. The root length was measured as shown in **(Fig. 1C)**. Different letters indicated significantly difference at *P* < 0.01. **(B)** Relative root length was measured as shown in **(Fig. 1C).** **(C)** Three-day-old *iar4-7*, *iar4-8* and WT were transferred to MS medium containing NaCl with various concentrations for 7 d. The root length was measured as shown in **(Fig. 1D)**.


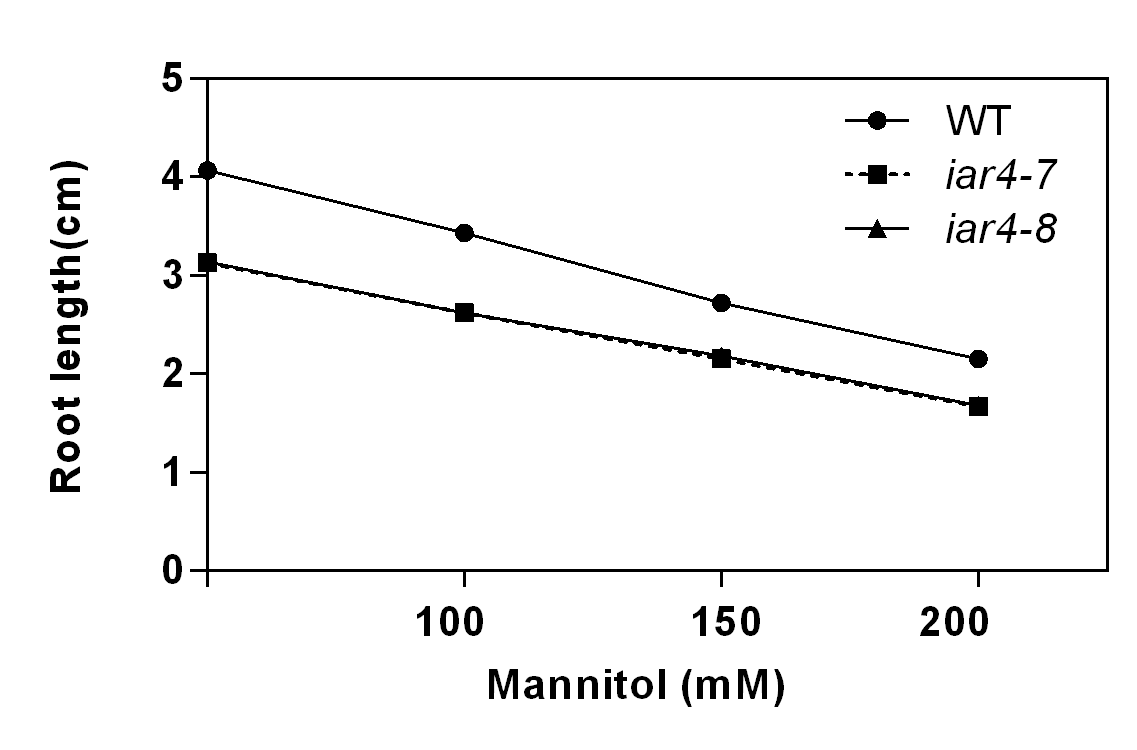


**Figure S3 |** Primary root growth under mannitol stress conditions. Three-day-old *iar4-7*, *iar4-8* and WT were transferred to MS medium supplemented with mannitol for 5 d. The root length was measured as shown in **(Fig. 2A)**.


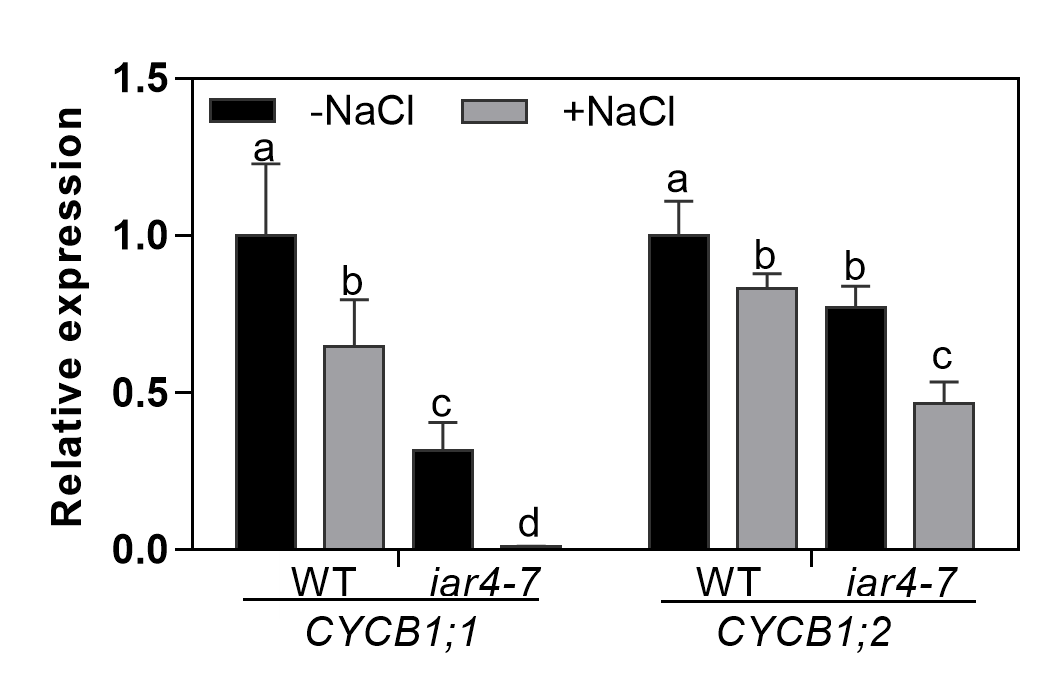


**Figure S4 |** *iar4* mutation reduced the cell division activity under salt stress conditions. Quantitative RT-PCR analyses of cell cycle marker genes (*CYCB1;1* and *CYCB1;2*) in roots treated with NaCl or not for 8 h.


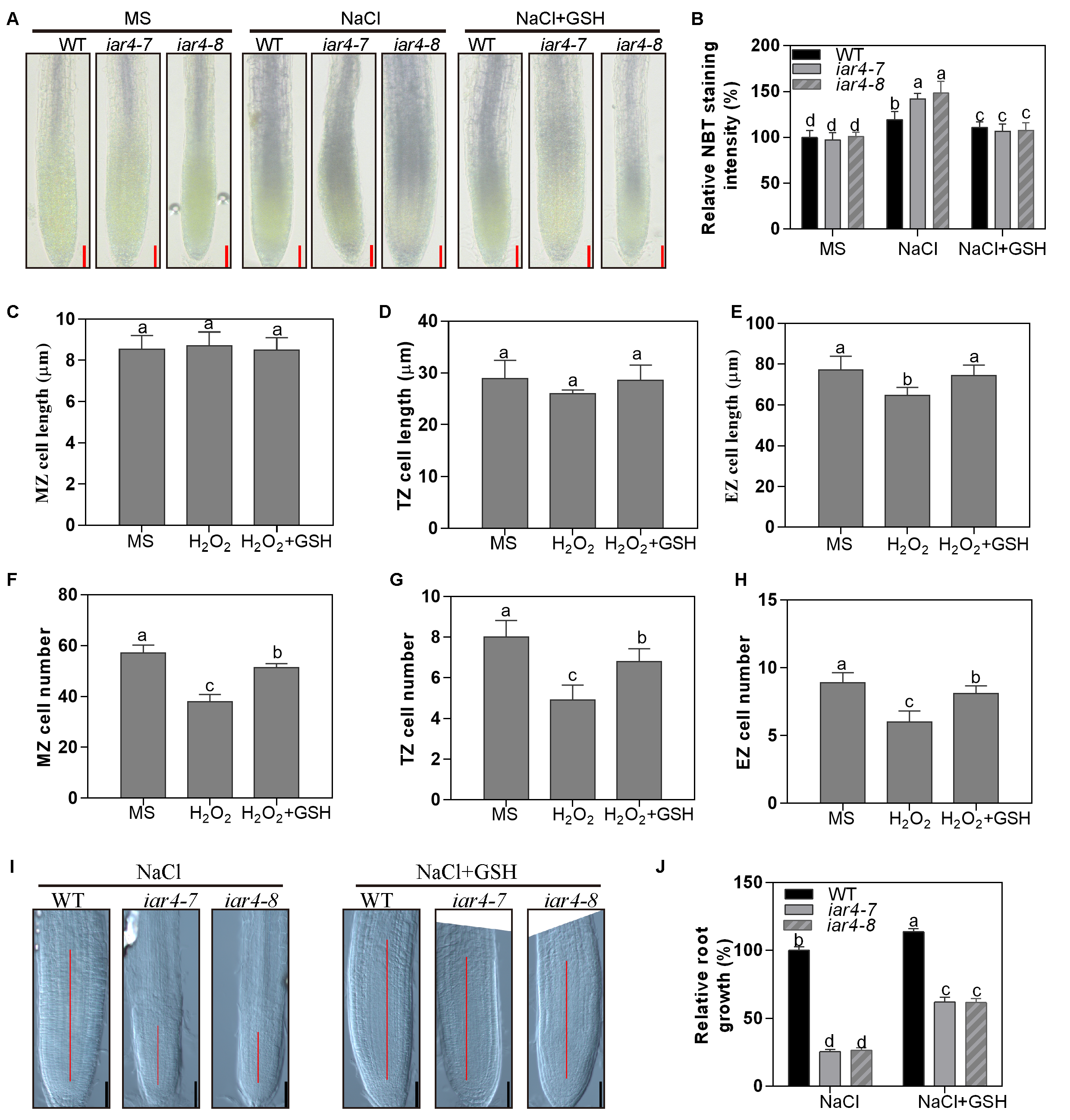


**Figure S5 |** GSH treatment partially reduced ROS levels and recovered the root length of *iar4* mutants under salt stress conditions. **(A)** NBT staining for superoxide in primary root of *iar4-7*, *iar4-8* and WT with NaCl or NaCl plus 200 µM GSH. Bars = 50 µm. **(B)** NBT staining intensity in *iar4-7*, *iar4-8* and WT with NaCl or NaCl plus 200 µM GSH was determined by Image J in **(A)** respectively. The intensity in WT without NaCl treatment was taken as 100%. Three independent experiments were done with similar results, each repeat with 10 roots. Data presented were means ± SD. Different letters indicated significantly difference at *P* < 0.01. **(C-E)** The MZ, TZ and EZ cell lengths of WT under 0.5mM H_2_O_2_ treatment with or without 200 μM GSH. Different letters indicated significantly difference at *P*<0.01. **(F-H)** The corresponding cell number of MZ, TZ and EZ under 0.5mM H_2_O_2_ treatment with or without 200 μM GSH. Different letters indicated significantly difference at *P* < 0.01. **(I)** Size of the amplified root meristem in *iar4-7*, *iar4-8* and WT under 100 mM NaCl treatment plus GSH or not. Bars = 50 μm. Three independent experiments were done with similar results, each with three biological repeats. **(J)** Three-day-old of *iar4-7*, *iar4-8* and WT mutant plants grown on MS medium supplemented with 100 mM NaCl plus 200 µM GSH for 10 d. The root length was measured as shown in **(Fig. 5C)**. The root length of WT with NaCl treatment was taken as 100%. Different letters indicated significantly difference at *P* < 0.001.


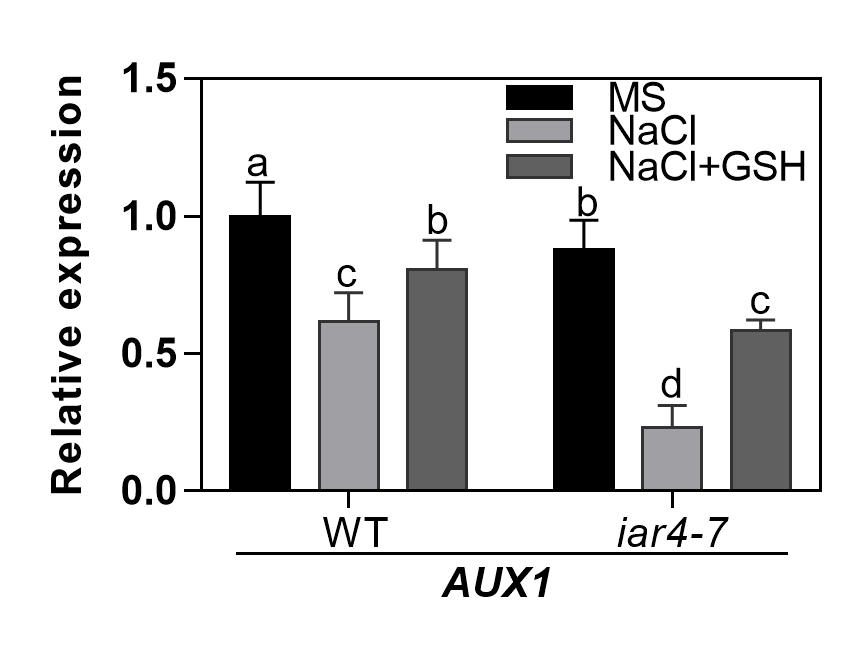


**Figure S6 |** *AUX1* expressions in *iar4* and WT under NaCl stress conditions with and without GSH treatment. **(A)** Quantitative RT-PCR analyses of auxin uptake gene (*AUX1*) in roots treated under NaCl stresses with or without GSH treatment for 8 h.


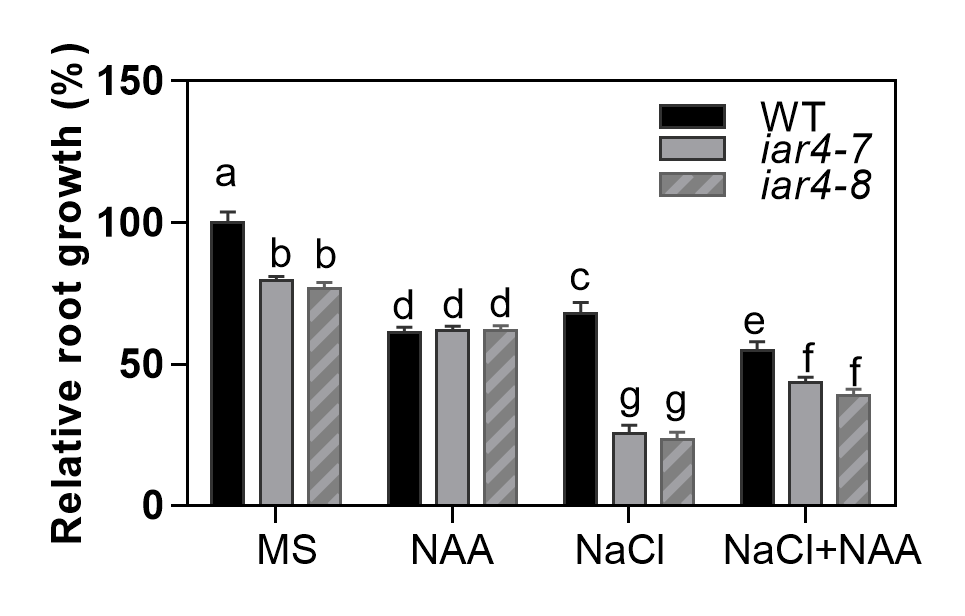


**Figure S7 |** Exogenous NAA largely restored the salt-inhibited root growth of *iar4* mutants. Three-day-old of WT and *iar4-7*, *iar4-8* plants were grown on MS, MS supplemented with 100 mM NaCl or 0.1 µM NAA or both for 10 d. The relative root length was statistically analyzed as shown in **(Fig. 7B)**, root length was expressed relative to that of the WT on MS medium without any treatment. Different letters indicated significantly difference at *P* < 0.05.


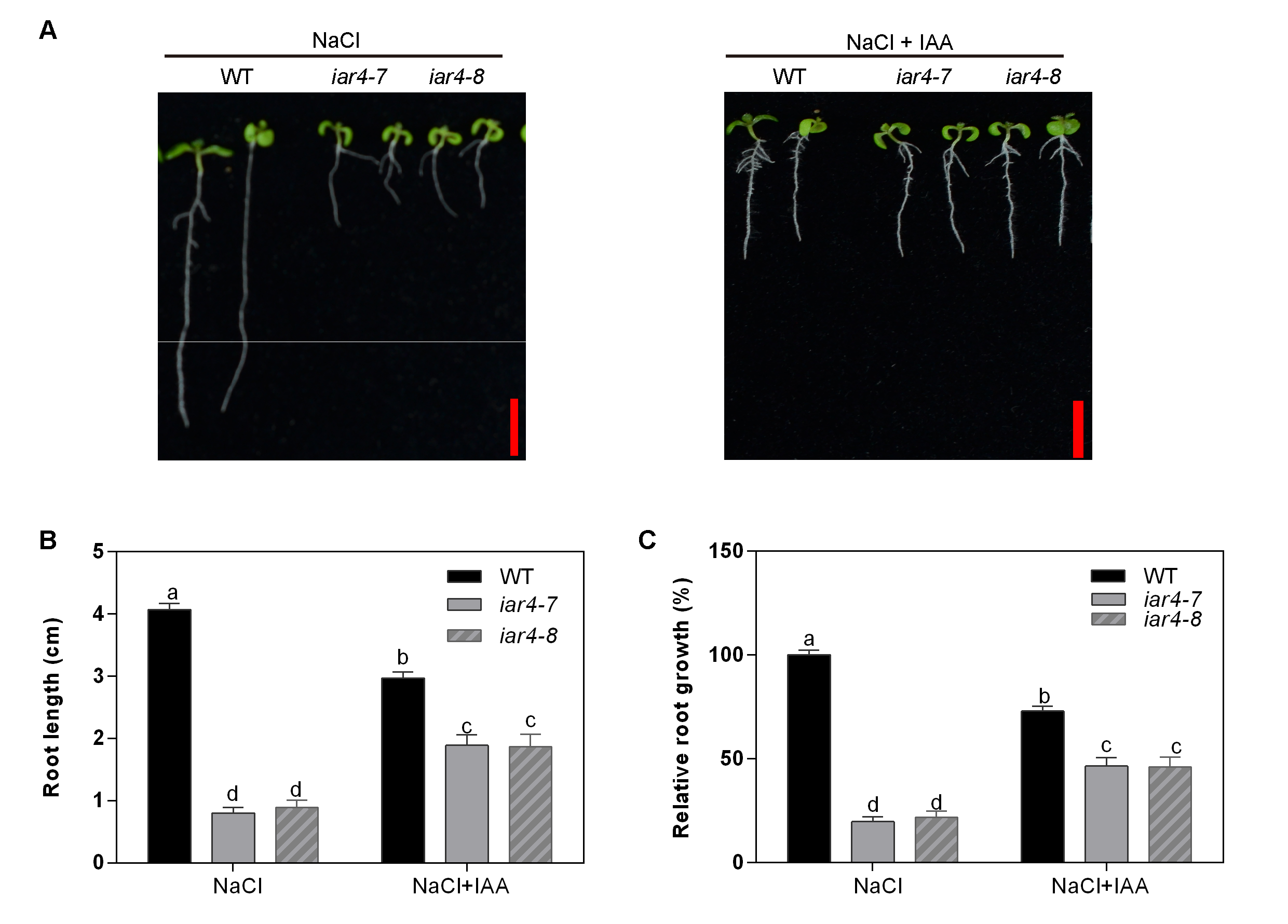


**Figure S8 |** Exogenous IAA largely restored the salt-inhibited root growth of *iar4* mutants. **(A)** Three-day-old of WT and *iar4-7*, *iar4-8* on MS, MS supplemented with 100 mM NaCl or 0.1 µM IAA or both for 7 d. **(B)** The root length was measured as shown in **(A)**. **(C)** The relative root growth rate of WT and *iar4-7*, *iar4-8* in **(B)**, root length was expressed relative to that of the WT on MS medium with 100 mM NaCl treatment. Different letters indicated significantly difference at *P* < 0.05. Scare bars are 1 cm.

**Table S1 |** Primers used in this study.

| Gene | Gene  reference | Use | Forward primer | Reverse primer |
| --- | --- | --- | --- | --- |
| *IAR4* | AT1G24180 | *iar4-7*  genotyping | GAGACCAGCACTTGAAAGCAC | AATCCCACAAAGCCGAAA |
| *IAR4* | AT1G24180 | *iar4-8*  genotyping | TATGTTCCTGGCTTGAAGGTG | CTGTCTCACGCCAGAGATTTC |
| *IAR4* | AT1G24180 | Complement-ation | GCTGGGCCCGCGAAGGTGAGGCTGAAT | AGTGGATCCCCGACGCAGAAGTTGGTT |
| *ACTIN7* | AT5G09810 | RT-PCR | GGCCGATGGTGAGGATATTCAGCCACTTG | TCGATGGACCTGACTCATCGTACTCACTC |
| *IAR4*  *AUX1* | AT1G24180  AT2G38120 | RT-PCR  qRT-PCR | AGGTGGATGGTATGGATGC  GACGCACTTCTCGACCACTC | GGAAGCAAGATTCTATGGAAGT  CCCAATCACTTTCTCCCACA |
| *CAT1*  *CYCB1;1*  *CYCB1;2* | AT1G20630  AT4G37490  AT5G06150 | qRT-PCR  qRT-PCR  qRT-PCR | GCCCCTAAATGTGCTCACC  GGAGGATAATCTCAAAAAACC  TACATTGCAGTTCCACACCGGCTA | AAGCACTTCTCACGATTTCCA  TCGAGCAGCAACTAAACCAAG  TAGCAACACCTCCATTCTCTGCCT |
| *GST1* | AT1G02930 | qRT-PCR | TCATCCTTCGCAACCCC | GCTATGATCGCCATGTCCTT |
| *HKT1* | AT4G10310 | qRT-PCR | GAAAGGCAAAATCTACAACGTG | CCTGCAAACCCATAACTCG |
| *NHX1* | AT5G27150 | qRT-PCR | GTGCTGTATCTATGGCTCTTGC | GGTAGCTTATGAGTGGTTTGGTC |
| *RBOHD* | AT5G47910 | qRT-PCR | GTACACCCACCATTTGTTCATC | AAAGCACGGAGCAGCCT |
| *RBOHF* | AT1G64060 | qRT-PCR | GACACGCCAAGACGAAAGA | ACACCCCGTTGGTCAAGTT |
| *SOD1* | AT3G10920 | qRT-PCR | GAACCTTGCTCCTTCCAGTG | TCTTCAGTTCTTTGTCTAGTCCG |
| *SOS1* | AT2G01980 | qRT-PCR | CTGGGAAGCCATATCTGTGC | GGACGCAAGAGTTTGAGAAGA |
| *UBQ5* | AT3G62250 | qRT-PCR | CGTGGTGGTGCTAAGAAGAGG | GAAAGTCCCAGCTCCACAGGT |
